# Vitreous Tamponades in Highly Myopic Eyes

**DOI:** 10.1155/2014/420380

**Published:** 2014-06-02

**Authors:** X. Valldeperas, J. Lorenzo-Carrero

**Affiliations:** ^1^Ophthalmology Department, Hospital Universitari Germans Trias, 08916 Barcelona, Spain; ^2^Ophthalmology Department, Hospital Povisa, 36211 Vigo, Spain

## Abstract

The use of endotamponade agents has gained a major role in the management of macular complications of high myopia. Myopic foveoschisis and macular hole are the main macular complication of pathologic myopia, this growing condition that is a main cause of visual loss, especially in patients at a younger age. We discuss the physical properties and advantages and disadvantages of the main ocular tamponade agents used in the treatment of these diseases.

## 1. Introduction

Intraocular tamponade agents are used to prevent the flow of intraocular fluid through retinal breaks, maintaining a temporary retinal attachment and allowing a persistent chorioretinal adhesion to appear after retinopexy is applied (laser photocoagulation or cryotherapy). Different endotamponade agents have been classically used: room air, sulphur hexafluoride (SF_6_), perfluoropropane (C_3_F_8_), and silicone oil. More recently, high-density or heavy silicone oils (HSO), a mixture of semifluorinated alkanes and silicone oil, have been described to provide tamponade to the inferior retina.

The effectiveness of a tamponade agent depends on its ability to maintain contact with the retina while displacing displacing the aqueous from the retinal surface. Several physical parameters, such as specific gravity, buoyancy, interfacial tension, and viscosity, influence this property [1].

The use of internal tamponades, specially gas agents, requires postoperative positioning in order not only to achieve good postoperative apposition between the bubble and the retina but also to avoid postoperative complications. Poor compliance with head positioning may potentially reduce the anatomical success rate.

High myopia, defined as a refractive error of >−6,00 D and an axial length of >26 mm [2], is a growing condition in developed countries, especially in Asia. These eyes can develop specific pathologies, such as myopic foveoschisis and retinal detachment (RD) secondary to macular hole (MH), that may need the use of an internal tamponade. Other common complications, such as rhegmatogenous RD due to peripheral retinal pathology, can also appear more frequently.

## 2. Indications of Endotamponade Use in High Myopic-Related Pathology

### 2.1. Retinal Detachment Secondary to Macular Hole

RD secondary to MH in highly myopic eyes is a challenging condition and one of the most difficult retinal detachments to treat. There is no clear understanding of the pathogenesis of myopic MH and RD, but anteroposterior and tangential traction from the posterior cortical vitreous, similar to idiopathic MH, have been suggested as the main causative factors [3]. Posterior staphyloma, causing inverse traction of the macula, and poor retinal adhesion secondary to retinal pigment epithelium (RPE) atrophy have also been described in the development of this condition [4].

Several treatment options have been proposed and, before the advent of pars plana vitrectomy (PPV), episcleral macular buckling alone was the standard of care in this situation. More recent approaches include the injection of an expansible gas bubble with or without PPV. Internal limiting membrane (ILM) peeling has also been implemented in the surgical procedure of these eyes in the last years.

Some authors have reported good outcomes after a more conservative approach, such as pneumatic retinopexy [5–7]. This surgical technique consists of injecting a gas bubble in the vitreous cavity, as internal tamponade, and prone position of the patient. In some cases, subretinal fluid is released through a sclerotomy [5, 6]. The best results reported with this technique are with RD involving only the posterior pole, provided that vitreous traction is absent [6]. Nevertheless, these studies were carried out before the optical coherence tomography (OCT) era, thus, making it difficult to assess the macular status of the patients. More recently, Ripandelli et al. reported a high retinal reattachment rate in a group of patients with RD due to MH and complete posterior vitreous detachment, treated with external drainage, pneumatic retinopexy, and transpupillary diode laser [7]. The treatment consisted of an injection of 1.5 to 2.5 cm^3^ of sterile 18% SF_6 _into the vitreous cavity via the pars plana under topical anaesthesia and face-down positioning for less than 7 days.

Li et al. performed a prospective study comparing the efficacy of simple intravitreal gas injection versus PPV combined with intraocular gas tamponade, for the treatment of RD secondary to MH in high myopia [8]. PPV with C_3_F_8_ endotamponade resulted in a higher anatomical success rate than intravitreal C_3_F_8_ gas injection alone. Though being inferior, pneumatic retinopexy resulted in 59.8% success rate after 6 months, therefore providing a good economic option in some cases. The authors reported no intra- or postoperative complications with pneumatic retinopexy. No retinopexy to the MH rim was used and, though patients underwent regularly OCT examinations, the MH closure was not reported.

PPV with the use of an endotamponade agent is the most commonly used technique for the management of RD secondary to MH in highly myopic eyes [3, 4, 9–16]. It is controversial, though, whether any of the available internal tamponades are associated with a higher retinal reattachment rate. What seems clear is that initial reattachment, within the first surgery, is correlated with a better final visual outcome [4].

Unfortunately, there are no randomized clinical trials comparing which tamponade agent yields the best outcome in these patients. Some authors have retrospectively compared different gas endotamponade agents with contradictory results. Uemoto et al. reported a higher rate of retinal reattachment and MH closure with C_3_F_8_ compared to SF_6_ gas [9]. Nakanishi et al. did not observe significant differences between types of gas tamponade. Interestingly, both studies are retrospective, and it is difficult to determine whether the duration of the gas tamponade may influence the surgical outcome [4].

The rationale for using a longer-action endotamponade in highly myopic eyes with MH and RD is to increase retinal reattachment rate and final visual outcome. This is based on the idea that shorter-acting gas does not provide a long-enough tamponade effect to allow for a glial reaction responsible for the closure of the MH and posterior retinal reattachment. This is especially relevant in highly myopic eyes, where the chorioretinal adhesion may not be as firm as it would be in patients with a healthy retinal pigment epithelium [9].

The repair of RD resulting from a posterior staphyloma-associated MH in highly myopic eyes may need more prolonged internal tamponade, as that given by silicone oil, in order to achieve MH closure and subretinal fluid reabsorption. Silicone oil in this situation shows additional advantages: shorter duration of prone positioning, faster visual rehabilitation, and easier follow-up of the retina and MH by the ophthalmologist [14]. Additionally, high myopic patients may benefit from the hyperopic shift induced by the refractive index of the silicone oil [17].

Scholda et al. reported a reattachment rate of 100% in eleven patients using silicone oil (5,000 centistokes (cSt)). The authors argued that silicone oil served as an inductor scaffold for glial closure of the causative macular hole. No additional manoeuvre was performed and the internal retina was left untouched. They did not remove the subretinal fluid through the macular hole. However, in this study, the authors did not use an OCT to assess the status of the macula after the surgery [13]. It is mandatory to achieve a complete closure of the macular hole in order to avoid recurrent retinal redetachment. OCT helps to predict anatomic and functional outcomes of highly myopic eyes having macular hole-related retinal detachment.

Silicone oil removal can be performed when MH closure is confirmed by OCT, while, in eyes with nonclosed MH, silicone oil removal may lead to a recurrent retinal redetachment [18].

#### 2.1.1. Myopic Posterior Staphyloma

Despite the documented advantages favouring silicone endotamponade, a recent study comparing silicone oil tamponade with C_3_F_8_ reported better results when C_3_F_8_ tamponade was employed. The initial rate of MH closure was 94% in the C_3_F_8_ group and 54% in the silicone oil group [19]. The authors point to the tendency for silicone oil to bridge across the margins of the staphyloma as one of the causes for higher rate of failure in the silicone group. It is well accepted that silicone oil bubble does not fit well into small recesses, such as the retina under the scleral buckling indentation, as well as into posterior staphyloma [20]. Interestingly, all subjects with initial anatomical failure achieved stable retinal reattachment after being reoperated with vitrectomy and HSO tamponade. Unfortunately, this is a small retrospective series and no definite conclusions can be established [19]. According to Nakanishi et al., the depth of posterior staphyloma may be associated with MH closure and RD reattachment rates, as it is difficult for the tamponade to fit into this area [4]. This is secondary to the buoyancy of silicone oil (specific gravity of 0.97 gr/mL) that is not enough to fit the posterior staphyloma, where there is practically no tamponade effect of the silicone oil. This raises an interesting question: why does MH in the presence of posterior staphyloma in highly myopic eyes close with silicone oil tamponade? Being a hydrophobic fluid, a thin layer of aqueous separates the silicone oil bubble from the retina. This is more evident in the presence of a posterior staphyloma in highly myopic eyes, where silicone oil does fit well and probably leaves a pocket of aqueous fluid. Fluid filled retinal areas experience negligible shear forces [21]. Besides, this compartmentalized fluid is scarcely influenced by ocular movements, and therefore fluid current is very low, generating even a lower shear retinal stress. This, in turn, may allow the MH to close and the RD to reattach.

#### 2.1.2. Is Prone Positioning Always Needed?

The role of postoperative posturing after vitreoretinal surgery is still controversial, as there is insufficient scientific evidence of whether it has a direct relationship with surgical outcome [14, 21]. A recent noncomparative study showed a high retinal reattachment rate performing PPV with internal limiting membrane peeling and silicone oil tamponade without any postoperative position restriction [14, 15]. Nevertheless, nonposturing surgery critically relies on the tamponade fill of the eye, especially in the early postoperative time [22]. This is even more important when silicone oil is used, because the typical round shape of the bubble it forms needs almost complete fill to make an effective contact with the retina. Complete vitrectomy and the greatest percentage of fill are always advisable to achieve maximal tamponade effect. It is important to note that tamponade efficiency does not depend on the size of the eye [22].

In the case of MH in highly myopic eyes treated with silicone oil as endotamponade, there should be no theoretical difference in whether standard or heavy silicone oil is used and whether the patient maintains a strict head posturing after the surgery, because it is a matter of low shear stress that closes the MH.

#### 2.1.3. Heavy Silicone Oils (HSO)

New HSO are mixtures of silicone oil with semifluorinated alkanes that combine a good tolerance with a satisfactory inferior tamponade. Oxane HD (Bausch & Lomb Inc., Waterford, Ireland) and Densiron (Fluoron GmbH. Neu-Ulm, Germany) were developed as vitreous substitutes to provide better inferior tamponade in cases of complicated retinal detachments with inferior breaks and proliferative vitreoretinopathy. HSO have a higher specific weight than water, which enables effective tamponade of the inferior retina, allowing the patient to adopt a supine posture postoperatively.

There are 2 studies comparing standard silicone oil and Densiron in highly myopic eyes with MH RD with different results. Avitabile et al. reported a better anatomical success rate with Densiron than 1000 cSt silicone oil [3]. Retinal redetachment after initial surgery with endotamponade in situ occurred more frequently in eyes filled with standard silicone oil, as all the eyes filled with Densiron had attached retinas. This finding was the same after removal of the silicone oil. In this study, patients did not get a significant visual improvement despite anatomical success probably due to the damage induced by the laser burns around the macular hole. In contrast, Mete et al., in a retrospective study, showed no statistically significant difference in retinal reattachment rate between eyes treated with standard silicone oil and Densiron [16].

Other authors have shown good results after using the combination of HSO and standard silicone oil in the treatment of RD with breaks and proliferative vitreoretinopathy involving the upper and lower quadrants [23].

#### 2.1.4. Disadvantages of Silicone Oil Endotamponade

One of the main downsides of using silicone oil as an ocular tamponade is the need for a second surgery to remove the oil. Another issue associated with silicone oil is intraocular pressure problems, sometimes related to oil emulsification [14]. Silicone oil viscosity has been classically proposed as the main factor affecting its emulsification. More recently, though, complete eye cavity fill and the presence of a scleral buckling have been described as factors even more important than viscosity influencing silicone oil emulsification. The presence of an encircling scleral element prevents emulsification by reducing the velocity of the oil inside the eye and therefore the shear force that generates emulsification [24]. Tamponade effectiveness of silicone oil is directly associated with the emulsification of this intraocular agent.

Comparing significant emulsification (e.g., abundant droplets of silicone oil in the anterior chamber or in the angle) of different types of silicone, Avitabile et al. found that, within 12 weeks of surgery, it was present in 30% of myopic eyes in the 1000 cSt silicone group and in 13% of the Densiron group. Minor dispersion of oil was also more frequently detected in the silicone group [3].

Other side effects described with either standard silicone oil or HSO are corneal opacity, corneal decompensation, and cataract formation. Others more specifically associated with Densiron are pseudohypopyon, due to intense emulsification, and chronic hypotony [25]. Inflammatory response may be another concern when using silicone oil. In a study comparing HSO with 1000 cSt silicone oil, Densiron showed a more proinflammatory profile [3]. Up to 40% of myopic eyes with retinal detachment treated with PPV and Densiron tamponade showed signs of inflammation, such as fibrin accumulation, keratopathy, or anterior chamber reaction. This inflammatory reaction was more frequent and intense than that seen in eyes treated with standard silicone oil. Interestingly, when managed with topical steroid therapy, they needed almost 1 week of treatment to control the inflammatory signs whereas eyes treated with standard oil responded in few days.

Unresponsive granulomatous inflammation, which usually resolves after HSO removal, has been reported with the use of HSO [26]. However, as suggested by Cheung et al., this finding might be secondary to the direct perfluorocarbon-HSO exchange [15]. Similarly, Veckeneer et al. have described an abnormal silicone oil adherence to the retina at the time of removal, related to the use of perfluorocarbon [27]. Other authors have confirmed these findings in vitro and related the “sticky oil” formation to the variation in temperature of the oil [28].

Sudden visual loss after silicone oil removal has been reported by several authors. Visual acuity drop can be severe and irreversible. It is not associated with other complications and it may happen after macula on retinal detachment. Fundus examination, fluorescein angiography, and OCT findings are usually unaltered, and only electroretinogram testing shows a different range of abnormalities, usually a severe macular dysfunction. There are several theories, but exact pathogenesis is unclear [29, 30].

### 2.2. Myopic Foveoschisis

Foveoschisis, in the presence of posterior staphyloma, is a major cause of visual impairment in highly myopic eyes. This condition has also been called macular retinoschisis, posterior retinoschisis, foveal retinoschisis, or shallow detachment of the macula, and its prevalence has been reported in up to 34% of eyes with pathologic myopia [31]. Foveal detachment is frequently associated, between 32% and 72% of the cases [32], complicating the situation and giving a lower visual acuity to the patient.

The pathogenesis of myopic foveoschisis and foveal detachment still remains unclear. Different factors have been related to its progression, but vitreous and epiretinal traction of residual vitreous cortex in the presence of a posterior staphyloma has been postulated as the main one. Other factors, such as poor elasticity or excessive rigidity of the ILM, stiffness of retinal vessels, progressive posterior staphyloma, and choroidal atrophy, may also play a relevant role in the pathogenesis [31, 33–37].

Some authors believe that this condition is not a true retinoschisis, with separation between retinal layers, but instead a form of retinal oedema secondary to vitreoretinal traction, and they have named it myopic traction maculopathy [38].

Natural course of myopic foveoschisis is variable, but it can remain stable for many years, without significant variation in visual acuity, with changes only appearing slowly over time [39, 40]. There is a report of spontaneous anatomical reattachment and visual improvement after posterior vitreous separation [41]. Nevertheless, other authors have described this condition as the initial step to the onset of a MH or a RD, in almost 50% of the patients [31, 33, 36, 39, 42, 43].

PPV, with or without ILM peeling, is widely accepted as the standard of care for macular schisis in high myopia [44–46]. Nevertheless, complications can occur, especially in patients with pathologic myopia: vitreous or macular haemorrhage, macular hole, ocular hypertension, and retinal breaks with or without retinal detachment. Furthermore, visual improvement is not always achieved after the macula has been reattached.

Other less invasive alternatives have been described. Anatomical success can be accomplished after gas tamponade without vitrectomy, although it may occur after a prolonged time period and multiple gas injections [32]. An intravitreal injection of C_3_F_8_, followed by prone positioning for 5–7 days, initially resolved 50% of cases with additional cases being resolved after repeated injections. According to the authors, this procedure is not suitable for cases with obvious central vitreomacular traction [32].

#### 2.2.1. Is Intraocular Tamponade Always Necessary?

Some studies have also questioned the necessity of using gas tamponade in patients who undergo PPV for myopic foveoschisis. Several authors have found that PPV with ILM peeling without gas tamponade results in resolution of foveoschisis and foveal reattachment, with an improvement in best-corrected visual acuity (BCVA) [38, 47]. The rationale behind this management is that the simple release of vitreoretinal traction without using any tamponade can slowly reverse macular distortion and lead to stable retinal anatomy restoration [38]. Additionally, this approach would be more favourable for the patient, as it would not require any postoperative positioning.

There are no accepted and universal criteria for vitreoretinal surgeons of when to use an intraocular tamponade in myopic foveoschisis. For Zheng et al., in a retrospective study, the criteria for using C_3_F_8_ or balance-salt solution (BSS) at the end of the PPV were exclusively based on the surgeon's experience and the feeling of where “macular region looked mobile and detached during posterior vitreous removal and ILM peeling” [33]. These authors showed a significant higher visual improvement when gas tamponade was used (logMAR BCVA change 0.58 ± 0.44) compared to the BSS group (logMAR BCVA change 0.25 ± 0.34). It is important to highlight that the presence of foveal detachment and duration of symptoms were not taken in account. Besides, final visual acuity could depend on the dye used for ILM peeling and time of incubation of the dye.

Uemoto et al. support the fact that long-acting gas endotamponade, like C_3_F_8_, even gives a better outcome than SF6, in the management of this condition [9].

By contrary, Kumagai et al. did not find a significant correlation between gas endotamponade use and final BCVA, in cases of myopic foveoschisis treated with PPV and ILM peeling [35]. Eyes treated with gas, though, showed a tendency to have better visual outcome. For these authors, the presence of a foveal detachment, in the preoperative period, was the best predictor for a better final BCVA [35].

#### 2.2.2. Does Intraocular Tamponade Induce Macular Holes in Eyes with Myopic Foveoschisis?

Several studies have pointed out the potential relationship between intravitreal tamponade and the creation of a MH, in cases of foveoschisis with foveal detachment in highly myopic eyes. Hirakata and Hida suggested that intravitreal gas might push the subretinal fluid inside the limited space under the foveal detachment toward the thin fovea, breaking this weak point and creating a MH [36]. These authors described a postoperative MH in 19% of eyes treated with endotamponade, all of which had a concomitant foveal detachment.

In a retrospective study, Kim et al. described the development of a MH in 22% of patients with foveoschisis and foveal detachment when gas tamponade was used, but no cases of MH were found in the group without tamponade [34]. On the contrary, Kumagai et al. described no cases of MH or other complications, in a retrospective study with 34 highly myopic eyes, after PPV, ILM peeling, and SF_6_ endotamponade [35]. Other authors have shown similar results using C_3_F_8_ [33]. Interestingly, Panozzo and Mercanti showed 25% of patients with myopic foveoschisis who developed a MH after PPV, when no endotamponade agent was used [38].

Studies on this topic reveal contradictory results and are mostly retrospective. Besides, ILM peeling was performed in many of the cases. This may be a potential confounding factor for the appearance of a MH, because this technique, although it has proven its effectiveness, can induce MH in eyes with very thin foveola [34, 37]. Supporting this idea, Shimada et al. reported 0% MH formation rate after fovea-sparing ILM peeling compared to 16.7% of MH in the conventional ILM peeling group, for the treatment of myopic traction maculopathy [48]. It is also important to note that myopic eyes are more prone to MH, even when PPV is not performed. MH has been described in almost 20% of fellow eyes in patients with myopic foveoschisis and foveal detachment [36].

It is not totally clear how a bubble of intraocular gas can improve anatomical restoration in myopic foveoschisis. Several factors have been related to its mechanism of action. Firstly, the gas bubble can induce displacement of outer layer detachments, by making RPE and retina together. Facedown positioning could enhance this. Once the subretinal fluid is spread out of the subfoveal area by the bubble of gas, healthier RPE cells can more easily pump it out [8, 32, 49]. Other authors suggest that the gas bubble generates a dry environment in the macular area, which has the potential effect of accelerating the reabsorption of residual fluid in the retina. This, in turn, may benefit the delivery of oxygen and metabolites to the outer retina [8, 33, 37]. But the mechanical effect of the gas bubble can last for a maximum of 1 or 2 months, until it has been totally reabsorbed. Resolution of foveoschisis, in many cases, can easily take more than this time, making it more difficult to understand the precise mechanism of action of the gas [34].

In eyes with myopic foveoschisis in which PPV is not performed, the gas bubble may act differently. The intraocular agent may work by stretching the posterior vitreous hyaloid and weakening the vitreoretinal adhesion.

#### 2.2.3. Is Anatomical Resolution Faster with Gas Tamponade?

Several studies have found that gas tamponade leads to faster anatomical resolution of myopic foveoschisis, compared to not using a gas tamponade [33, 34]. Kim et al. showed that the mean time for resolution was 2.25 months (range: 1–3) for the gas treated group, whereas it was 4.50 months (range: 2–8) for the group without tamponade [34]. Similarly, Panozzo et al, in a noncomparative study, described a slow process of anatomical recovery (mean: 4.4 months; range: 1–12 months) [38]. The time needed for anatomical resolution for Zheng et al. was obviously longer in the BSS group (without tamponade) [33].

## 3. Conclusion

High myopia macular complications are an increasing cause of visual loss. New diagnostic technologies have greatly increased our understanding of these pathologies and help to plan the surgical approach that yields the best postoperative result. The use of endotamponade agents plays a definite role in the surgical management of these patients, especially with myopic foveoschisis and RD secondary to myopic MH. Comprehensive knowledge of the physical properties, indications and potential complications of the different available tamponade agents will help us to improve the care of our highly myopic patients.
